# The impact of investigator bias in nutrition research

**DOI:** 10.3389/fnut.2025.1513343

**Published:** 2025-04-17

**Authors:** Sangeetha Shyam, Jordi Salas-Salvadó

**Affiliations:** ^1^Alimentació, Nutrició, Desenvolupament i Salut Mental (ANUT-DSM), Institut d'Investigació Sanitària Pere Virgili (IISPV), Reus, Spain; ^2^Rovira i Virgili University, Departament of Biochemistry and Biotechnology, Human Nutrition Unit, Reus, Spain; ^3^Centro de Investigación Biomédica en Red Fisiopatología de La Obesidad y La Nutrición (CIBEROBN), Instituto de Salud Carlos III, Madrid, Spain

**Keywords:** bias, meta-research, research methodology, ethics, nutrition

## 1 Introduction

Public trust in nutrition science has waned over time in several countries (1, 2). Professional bodies persevere to reverse the trend or earn trust by improving rigor in research conduct and dissemination (3–5). While several biases in research are cataloged and remedial measures suggested, “the investigator bias” arising from investigators themselves, poses a major challenge. Investigator bias rarely receives mention in academic literature. There is currently no universally accepted definition or assessment for investigator bias. In 2003, Ernst and Canter in their short letter on investigator bias and false positive findings defined it as a “bias resulting from a conflict of interest arising from passionate beliefs held by the investigators” (6). We define “investigator bias” as acts of omission or commission that arise from ignorance, hubris or excessive attachment to beliefs, based on existing discourse (7, 8). In our viewpoint, we do not focus on research misconduct such as data falsification or unethical publication practices; as their perils are well-recognized and clear-cut mechanisms exist to prevent, and redress these concerns. Without a clear framework to either identify or assess investigator bias, it is unsurprising that there is a paucity of empirical evidence on the extent of its prevalence, or its impact or more importantly to address it.

The lack of formal evaluation of investigator bias is concerning given that investigator bias encroaches into one or more stages in the research process including study conception, design, conduct, analysis, interpretation and dissemination, challenging research credibility (6). It influences what research questions are posed, how hypotheses are tested and interpreted (6, 7). Such positional biases in reviewers and funders can have consequences for the field, by determining what is rewarded and perpetuated. To tackle bias, it would be imperative to understand what drives it. Opportunities and career advancement in academia and research are increasingly competitive and are linked to the ability to attract funding and publish in indexed journals with high impact. Funders and institutions are predominantly defined by “normal” science characterized by its stability, driven by set precedents, and with a tendency to lean toward the wisdom of accepted figures in the field (9). Therefore, questioning current practices and paradigms may be problematic and unproductive for researchers. Additionally, promotional language in grant applications is known to increase their chance of being funded and the citation impact of resulting publications from these grants (10).

Therefore, it is possible that grant applications or publications apart from reflecting the investigators' beliefs and hypotheses, may also be catering to what funders, journals or reviewers are known to value. It is important to note that it is impossible to personally know all authors to sufficiently discriminate between playing to the gallery and reflecting inherent biases in grants and publications. Thus, we are bound to interpret what is written and reported as a proxy for an author's personal biases. When biased grant applications or publications are successful, the success in itself reinforces current behavior in investigators, creating a feedback loop. Thus, current systems and practices lead to the perpetuation of the practice of investigator bias and its consequences.

**Figure 1:**
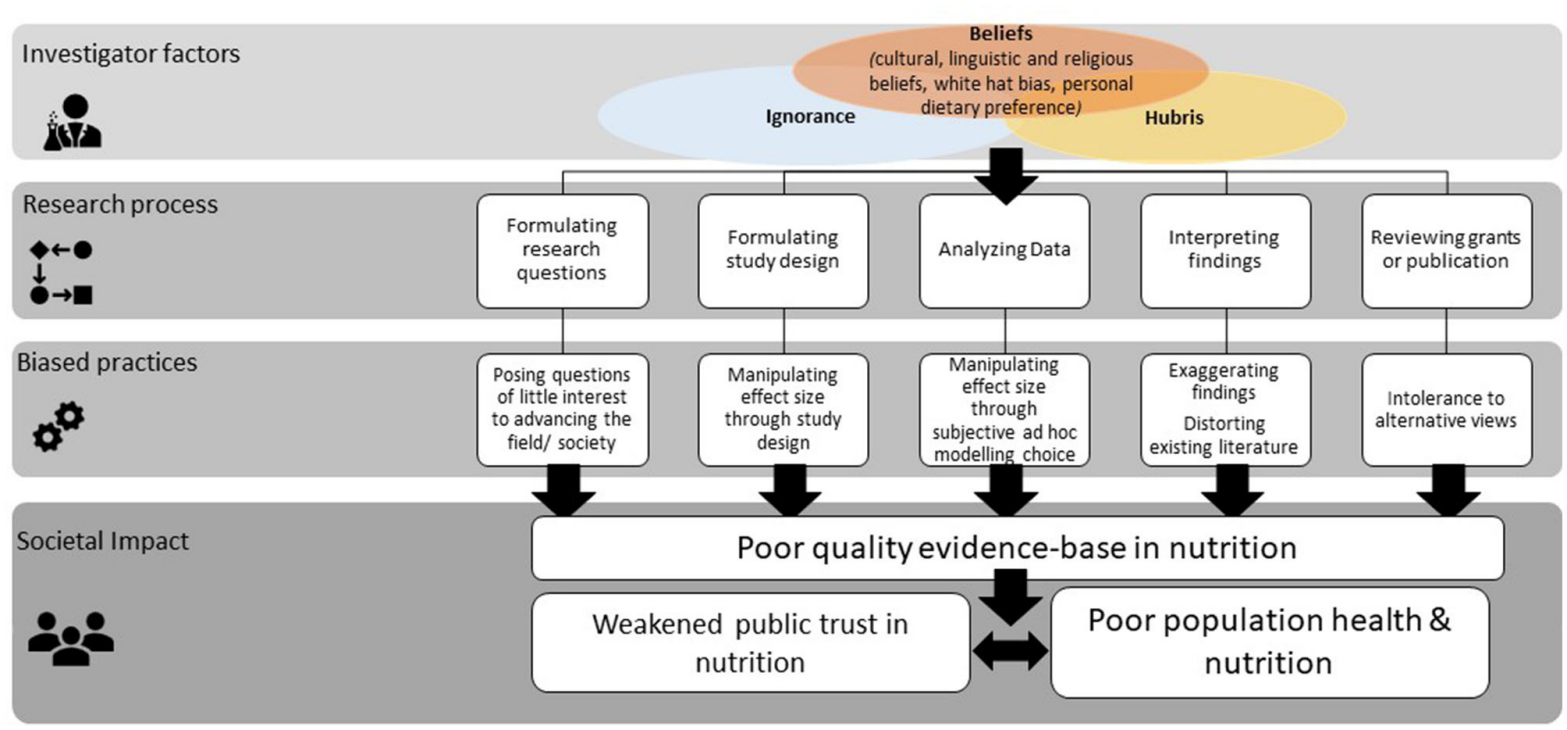
Consequences of investigator bias on public trust in nutrition. The figure shows the impact of personal factors influencing investigator bias, potential expression of investigator-bias at various stages in the research process and its consequence on public trust in nutrition.

Without a global understanding of investigator bias, it is impossible to confidently describe or quantify its presence and impact in nutrition. However, the discussion on dissonance between the cultural basis of food and the Eurocentric values of nutrition science (the formal study of food) over a decade ago (11), makes one ponder if investigator bias can affect nutrition in more profound ways than it does to other areas of science. As if in agreement, in 2018, Ionnadis and Trepanowski published a highly provocative opinion piece. The opening lines of the paper read: “nutrition research is among the most contentious fields of science” and the authors argued that non-financial conflicts of interest in nutrition were beyond “allegiance bias and preference for favorite theories” that exist in any field of study (12). The authors proposed that nutrition scientists faced additional challenges that arose from making personal dietary choices that were shaped by familial, cultural, or religious norms. The controversial perspective evoked passionate response from nutrition scientists. A counterpoint totally dismissed these notions (13). The Editorial board of the European Journal of Nutrition responded with an editorial that highlighted the “need for self-reflection” in scientists funded by the industry and those “seeking to prove their favored hypothesis” (14). Thus there exists some consensus that investigator bias in nutrition can misinform dietary guidelines, create disagreements among experts in the public domain, and further erode trust in nutrition (15) (Figure 1). Thus, it is both urgent and important to tackle this bias from the public health perspective. A potential first step to negate investigator bias and to effect change is to create awareness among all stakeholders. As investigator bias rarely features in current scholarly discussions in nutrition, we aim to create awareness, by providing a few field-specific examples of investigator bias and identify or propose some measures to address it (Table 1). The aim of this perspective piece is not to be an exhaustive reference of examples of investigator bias and recommended remedial measures. Through this piece, we aim to begin a dialogue with stakeholders of nutrition research (which includes funders, ethics committees, academics, scientists, trainees, practitioners, and consumers), to create awareness, and galvanize efforts to appropriately tackle the bias.

**Table 1:** Examples of investigator bias and potential preventive measures at each stage of the research process.

**Step in the research process**	**Biased practice**	**Examples**	**Preventive measures**
Formulating research	Asking questions that add no value to extending understanding of nutrition or societal impact.	#x02022; Repetitive systematic reviews that have no added value (39). • Focus on a single/narrow set of intermediary outcomes that do not explain a primary outcome of interest • Asking questions that will not alter current practice driven by white hat and/or positional biases	#x02022; Including patients, non-researchers and external experts in the field of study • Checking trial and review registry databases for redundancy of proposed study • Establishing a study design board that vets study protocols for scientific validity, and ensures their appropriateness to proceed with ethics application to IRBs • Use of adversarial collaborative (38)/red team (37) approach to tackle white-hat bias • Pre-registration of protocols to emphasize the importance of the research question and the quality of methodology by conducting peer review prior to data collection (40) • Adequate evaluation by reviewers (e.g., study design board, funders etc.) on how the proposed research adds to/extends existing evidence). • Declaration of conflicts of interest of authors to include funding and non-financial interest disclosures • Declaration of conflicts of interest to include funding disclosures and positional statements for reviewers and editors • Ensuring diversity in editorial boards and review panels
Formulating study design	Asking questions in a way to reach a certain end newline Adopting methods unsuited to answer the research question of public interest	#x02022; Manipulating effect size through using inappropriate control (23) • Bundling interventions that do not allow for teasing out the independent variable of interest (24) • Selection bias (27) • Applying inappropriate solutions from other populations without contextualization (41, 42)	#x02022; Use of adversarial collaborative (38)/red team (37) approach to tackle white-hat bias • Pre-registration of protocols to emphasize the importance of the research question and the quality of methodology by conducting peer review prior to data collection (40) • Establishment of a study design board that vet study protocols for scientific validity checks, and ensure their appropriateness for ethics submission to IRBs • Insistence by study approval boards, and funders on detailed submission of protocols for review • Inclusion of external experts in epidemiology, trial methodology, and nutrition to evaluate the scientific, cultural and ethical appropriateness of the study design aspects including the appropriate use of placebo or control.
Analyzing data	Manipulating data analytical techniques	#x02022; Subjective and ad hoc choice of models (27) to support white-hat and positional biases • P-hacking (30)	#x02022; Adoption of pre-specified SAP for primary, secondary and exploratory studies • Inclusion of justification of the choice of models in SAP • Provision of information relating to a priori SAP in the methods section of the manuscript (or acknowledging the lack of it and its potential limitation) • Identification of studies as exploratory or secondary analysis in titles and abstracts of publications
Interpreting findings	Inappropriate interpretation of results	#x02022; Exaggeration of small effects (33) • White hat bias while interpreting existing literature or study findings (34)	#x02022; Declaration of conflicts of interest to include funding disclosures and positional statements • Adequate peer review training to identify investigator-bias in grant applications and publications being reviewed. (This may also sensitize researchers to their own biases) • Incentivizing peer-reviewers to improve the quality of the peer-review
Evaluating and providing feedback	Inappropriate review of grants and publications	#x02022; Affirming work in alignment with personal beliefs and positional biases • Rejecting or excessively challenging alternative views to one's own beliefs	#x02022; Adequate peer review training to identify investigator-bias in grant applications and publications being reviewed. (This may also sensitize researchers to their own biases) • Incentivizing peer-reviewers to improve the quality of the peer-review • Declaration of conflicts of interest of authors to include funding and non-financial interest disclosures and • Declaration of conflicts of interest to include funding disclosures and positional statements for reviewers and editors • Ensuring diversity in editorial boards and review panels

## 2 Influence of investigator bias on nutrition research

### 2.1 Asking questions no one wants or needs answers for

Investigator bias can result in pursuing questions solely of interest to researchers, without optimal considerations of study impact or value addition (16). These pursuits can arise from a deliberate antagonistic positioning to established evidence in public health driven by “science-related populism” (17). This phenomenon increasingly seen in the West (17), has also been noted in nutrition and often adds to confusion and stalls decision-making and policy implementation (18, 19). At other times, biased pursuits include retesting globally well-proven hypotheses. While replication is an important aspect of rigor and validity in science, reproduction of an established concept without clear justification for the same leads to redundancy and resource wastage. For instance, while evidence synthesis is key to policymaking, performing yet another systematic review or meta-analysis without differentiation, just adds to clutter (20). Other trials, even when novel, are unlikely to change practice. For example, a trial comparing the effects of moderate alcohol consumption vs. abstinence, irrespective of its findings, is unlikely to change recommendations given the safety concerns.

### 2.2 Adopting inappropriate methods

Investigator bias can also affect the framing of a research question as there may be a tendency to “try to prove” a certain hypothesis. This may include conscious or unconscious manipulation of one or more of the Population, Intervention, Comparator, Outcome, and Time (PICOT) variables. For example, caution against the use of surrogate markers as endpoints has often been expressed (21). This concern especially bears weight in nutrition studies where the effects of diet tend to be chronic occurring over time; while feasibility, practicality, and funding make us settle for typically shorter study periods. The problem is compounded by investigator bias, when studies have outcomes (endpoints) that satisfy the researcher's curiosity and belief without sufficient consideration of its relevance or appropriateness. For instance, one's viewpoint on the benefit of high linoleic acid intervention or lack of it in comparison to saturated fat can be reaffirmed through the choice of using total low-density lipoprotein- (LDL)—cholesterol or oxidized LDL as the outcome of interest. While linoleic acid lowers vs. total LDL-Cholesterol, it increases oxidized LDL (22). Therefore, when viewed individually each of these outcomes may provide contradicting results.

Also, when investigators strongly believe or distrust an intervention, the treatment effect in clinical trials may be (un)consciously altered through manipulating the control. This especially happens when no “inert” placebo can be used (as in the majority of nutritional trials). For example, a randomized controlled trial to assess the effect of nuts on LDL-cholesterol could have any of these controls: (a) olive oil matched for added fat, (b) a fruit serving matched for added calories, and (c) a refined carbohydrate snack matching added calories and fat. However, each of these controls varies in their health effects and will yield different effect sizes. A refined carbohydrate snack while seemingly a good model to study the effect of nuts replacing a snack, is more likely to show nuts in a better light. Contrarily, given their potential health benefits, olive oil and fruits as control may decrease the difference in outcomes between study arms. On a similar note, it is often noted that behavioral intervention trials to improve health outcomes through effecting dietary changes often include controls reflecting a dismal standard of care, e.g., providing educational pamphlets vs. reflecting an ideal standard of care (23).

Another form of effect size manipulation occurs when researchers want to test a new behavioral intervention against the standard practice but create a difference in the intensity of delivery between arms. Frequent follow-up or even logging, increases adherence to behavioral interventions. Therefore, a more frequently or intensively followed-up intervention group performs better irrespective of intervention content. This was in part why the findings from a much-awaited personalized nutrition trial were disappointing to readers as the intervention bundling precluded singling out the effect of individualization from intensive intervention (24).

Additionally, investigators may apply solutions generated in a specific population to others without contextualization. Total diet replacements facilitating diabetes remission in the United Kingdom, for instance, may be impractical for India or Spain for different reasons. Again, building prediction models, which is expected to be increasingly used in nutrition (25), should desist from using available input variables in a dataset, without considering the feasibility of using these variables in the intended context. Such models are highly likely to fail to meet its intended purpose.

### 2.3 Manipulating analytical techniques

Observational studies are often criticized for reporting exaggerated associations between dietary risks and health outcomes (8), and frequently not corroborated results coming from randomized controlled trials (26, 27). The observed differences in effect estimates were found to be driven by heterogeneities in population, definitions of intervention, exposure, comparator, or outcomes (28, 29). Additionally, cohort studies were noted to be more prone to residual confounding, and the variables included in the statistical models had significant consequences for the direction and magnitude of associations estimated (29). Thus, it is likely that bias in nutrition research can arise from inappropriate modeling or biased covariate choices that inadequately control for confounding, or result in over-adjustment or failure to recognize unrecoverable selection bias. In this context, unspecified exploratory analyses are particularly vulnerable to investigator bias-related p-hacking, due to the lack of prior evidence for biological plausibility (30).

### 2.4 Inappropriate interpretation

While manipulation or subversion of negative results favoring industry is well established similar bias that occurs in the absence of industry collaborations are ignored (16, 31, 32). Obsession with pre-conceived ideas leads to exaggerated reporting of weak, statistically significant results without clear clinical relevance (33), creating unnecessary distraction. White hat bias, another investigator-linked bias displayed on strongly felt topics, arises from perceived action for righteous ends and leads to the distortion of research findings (34). White-hat bias has been previously identified in nutrition topics such as obesity, non-nutritive sweeteners, and breastfeeding (34, 35). More recently, white-hat bias has come to display as strong polarized views on ultra-processed food, some of which could also be driven by the populist media attention. Furthermore, over interpretation of results from observational studies assuming a cause-effect relationship is also frequently reported in literature (28, 29) and may arise from investigator bias.

### 2.5 Inappropriate evaluation and feedback

While there is some acceptance that positional biases in investigators, both implicit and explicit, can affect research evidence creation and reporting in several ways, its role in shaping the evidence-base for nutrition through evaluation and peer-review activities is even less understood or discussed. As a relatively recent science that emerged from medical sciences, nutrition continues to be bound by tendencies for reductionism and pharmaceutical modeling logics (11). Lack of sufficient cross-cultural engagement and poor understanding of the complex, holistic, cultural and social underpinnings of nutrition in evaluators, and reviewers is likely to lead to irrelevant feedback and inappropriate evaluation. As investigators also service the profession through peer-review and editorial activities, it is important to acknowledge that personal biases arising from disciplinary, cultural and/or linguistic basis have the potential to skew the existing literature. In the absence of diversity among reviewers and editors, publication bias could arise from the compulsion to conform to mainstream ideas and practices.

## 3 Conclusion: final thoughts and the way forward

Since we are but human, we are all touched by ignorance, hubris and beliefs, and therefore are not immune to investigator bias. Established expertise by default does not invite or encourage criticism. Therefore, the current focus of the research ecosystem on financial interests to address conflict of interest, with no clear mandate on managing non-financial interests (36), is inadequate to deal with the bias that can arise from investigators themselves and support good science. Moreover, reporting non-financial conflicts predominantly occurs after study completion, and during publication. To prevent the colossal waste of resources, attempts to identify investigator bias are needed earlier and throughout the research process.

Despite acknowledgment of investigator bias in nutrition, the existing nihilism in certain sections is defeatist and has prevented progress in addressing the bias. As a way forward, we propose the following suggestions. First, in the absence of punitive measures for the consequences of errors in nutrition research and practice, the first line of defense against investigator bias is self and peer regulation. Thus, the earlier we choose to acknowledge and sensitize ourselves to this bias that arises from within, the better prepared we are to prevent ourselves and our peers from falling prey to it. As a starting point for these discussions and to create awareness and encourage introspection and reflection within the nutrition research community, we have collated a few existing safeguards, suggested the repurposing of a few others or propose a few other aligned with steps in the research process in Table 1. Reflective practices, we believe, can result in a more thorough evaluation of the bias, and further deliberation on whether formal processes to recommend best practices to deal with the bias (such as developing assessment tools or checklists) are necessary.

Second, systematic and formalized collective efforts to define and catalog investigator bias and identify where the bias arises from are important, warranting dedicated task forces established by professional societies for the purpose. These efforts should preferably translate, as described earlier, to developing assessment tools to identify the bias, and therefore help quantify its presence and further enable studying trends over time. The task force could also deliberate and recommend appropriate ways to report positional biases (such as those arising from dietary preferences) in authors, reviewers, and editors. While mandating authors to declare conflicts of interest has received major attention in recent decades, it is also important to acknowledge and manage reviewer and editorial bias as their actions are definitive and determine how science is shaped.

Thirdly, a conscious approach to building tolerance to alternative views and improving diversity within research teams, Institutional Review Boards (IRB), editorial boards and reviewer panels should be encouraged at personal and institutional levels. Several of the issues discussed above, would have been prevented if red teaming/adversarial collaborative approaches were practiced (37, 38).

Finally, and importantly, being prescriptive or improving declaration of conflicts of interest has never been the panacea to eradicating biases. Therefore, emphasizing reflection on its occurrence and emphasizing this in training of younger generation of scientists may be the best bet yet.
